# Editorial: Injuries, injury prevention and training in climbing

**DOI:** 10.3389/fspor.2024.1390338

**Published:** 2024-03-14

**Authors:** Atle Hole Saeterbakken, Volker Rainer Schöffl, Andreas Schweizer, Gudmund Grønhaug

**Affiliations:** ^1^ Department of Sport, Food and Natural Sciences, Faculty of Education, Arts and Sports, Western Norway University of Applied Sciences, Sogndal, Norway; ^2^ Klinikum Bamberg, Sozialstiftung Bamberg, Bamberg, Germany; ^3^ Balgrist University Hospital, Zurich, Switzerland

**Keywords:** climbing, sports climbing, climbing injuries, injury prevention, chronic injuries, sports medicine

**Editorial on the Research Topic** Injuries, injury prevention and training in climbing

Centuries ago, climbing pioneers began exploring mountains and high peaks. With the introduction of indoor climbing centers, climbing as an activity has evolved into a sport. The 2024 Olympics in Paris will feature individual climbing competitions in bouldering, lead climbing, and speed climbing (1). New climbing gyms are opening every year in every major city. With growing popularity and increasing performance levels, a need for evidence-based knowledge on injury prevention, testing, and training has emerged (2, 3). In particular, climbing research is in its infancy, but the literature is expanding rapidly (4–7). However, there remains a need to improve knowledge about injury prevention strategies, injury epidemiology, and sports medicine, including systematic training approaches for returning to climbing post-injury. Therefore, the Research Topic “Injuries, Injury Prevention, and Training in Climbing” aims to advance scientific understanding in these areas.

Sixty-four authors from Europe and the Americas contributed to the 12 papers published in this Research Topic. Notably, half of the papers include authors from multiple countries, highlighting the importance of collaboration in filling knowledge gaps. The manuscripts vary in methodology: three studies utilized surveys, three conducted training or rehabilitation interventions, two were systematic reviews, and four used a cross-sectional design. Of note, four studies involved competitive or elite climbers, a group almost entirely absent from the scientific literature and whose inclusion has been called for. The interdisciplinary evidence of this Research Topic has multiple applications: (a) chronic injury prevalence rates, low back pain, (b) eating disorders, amenorrhea, and nutritional knowledge among competitive climbers, (c) the development of new training methods to potentially reduce injury rates, (d) finger diagnostics, (e) the testing and measurement of climbing performance, and (f) recovery and fatigue states after climbing.

Injury prevention is a part of all sports. Chronic injuries are often the result of high intensity over an extended period of time without adequate rest or recovery strategies. Carraro et al. examined the prevalence of low back pain in 180 competitive climbers aged 13–19 years. Over the previous 12 months, 74% had reported low back complaints, most of which were classified as low-intensity to low-disability (63%). Concerns over non-traumatic, overuse injuries in climbing have been previously addressed but not in a systematic review that examines potential risk factors and injury prevention strategies. Quarmby et al. included 34 studies in their review and identified higher climbing intensity, bouldering, reduced finger strength, use of the crimp grip, and previous injuries as risk factors for overuse injuries. However, findings related to gender, climbing experience, and training volume were inconsistent, while body weight/BMI, stretching, and warm-up/cool-down routines were not associated with an increased risk of injury. Concerning the potential for an exaggerated focus on body weight in climbing, injuries, amenorrhea, and eating disorders were assessed among 114 elite female competitive climbers (Grønhaug et al.). More than 53% reported injuries in the previous 12 months, with shoulders (38%) and fingers (34%) being the most common injury locations. BMI did not show an increased odds ratio for injury, but those with an eating disorder had twice the odds of being injured. In a study of 50 competitive boulderers in the UK, nutritional knowledge scores were average, with considerable individual variation (Gibson-Smith et al.). Moreover, 38% of female athletes and 46% of male athletes reported intentional weight loss, with 76% engaging in concerning practices. It is, therefore, crucial that trainers and professionals remain vigilant in identifying athletes at risk for problematic behaviors early, addressing the issue, and establishing appropriate specialist services.

Finger injuries are the most common affliction among climbers (8). Therefore, Gronhaug et al. compared finger cartilage composition using MRI between 13 climbers and ten non-climbers and found no significant difference in T2 values between the groups. Additionally, Bayer et al. examined the clinical management of finger joint capsulitis/synovitis in rock climbing through a case study. Following a 6-week comprehensive rehabilitation program that focused on unloading affected tissues, increasing mobility, correcting climbing movements, and improving muscle performance, pain levels decreased from 5.5 to 1.5 on the 0–10 analog pain scale. Furthermore, Devise et al. implemented a 4-week, twice-weekly hangboard training program and divided 52 experienced climbers into four groups. The study found that only the extensor-based training significantly improved finger extensor strength.

Exel et al. demonstrated that performing a dead hang with the arms fully extended placed less stress on the elbow and shoulder joints compared to elbow flexion at 90 and 135°. Moreover, Javorsky et al. compared two 5-week periods of low-volume blood flow restriction (BFR) training with high-intensity resistance training in intermediate climbers. The results showed that low-intensity BFR training (30% of maximum) yielded similar climbing-specific strength and endurance outcomes as high-intensity resistance training (60% of maximum), suggesting that BFR may be an effective alternative for reducing mechanical stress on the fingers.

In their systematic review, Langer et al. sought to provide an overview of the diagnostic tests and performance measurements in climbing. From 148 studies, 63 different tests were identified, indicating a lack of uniform or standard procedures for evaluating climbing performance. Assessment of recovery markers associated with climbing could potentially reduce the incidence of overuse injuries. Gasparie et al. analyzed a range of recovery markers 4 min post-competition, and 12, 24, 48, and 60 h following a national bouldering competition. They found that forearm strength and pain returned to pre-competition levels within 24 h, but climbing readiness was still compromised 48 h after the competition. In a separate study, Yu et al. reported that 24 h of continuous rock climbing led to a 15% reduction in mean grip strength and a 71% reduction in dead hang endurance in 36 climbers.

As we conclude this Research Topic, we are confident that we have addressed numerous gaps in evidence-based knowledge and significantly advanced the understanding of injury, injury prevention, and training in climbing. We trust that this compilation of studies will provide valuable and practical insights for both recreational and competitive climbers, as well as coaches. We also encourage researchers around the world to add to the body of evidence-based climbing literature.
